# Biocontrol of Phage Resistance in *Pseudomonas* Infections: Insights into Directed Breaking of Spontaneous Evolutionary Selection in Phage Therapy

**DOI:** 10.3390/v17081080

**Published:** 2025-08-04

**Authors:** Jumpei Fujiki, Daigo Yokoyama, Haruka Yamamoto, Nana Kimura, Manaho Shimizu, Hinatsu Kobayashi, Keisuke Nakamura, Hidetomo Iwano

**Affiliations:** 1 Department of Veterinary Medicine, Rakuno Gakuen University, Ebetsu 069-8501, Japanh-iwano@rakuno.ac.jp (H.I.); 2 Phage Therapy Institute, Comprehensive Research Organization, Waseda University, Tokyo 162-8480, Japan

**Keywords:** bacteriophage, evolved phages, infection control, fitness cost, trade-offs, antimicrobial resistance

## Abstract

Phage therapy, long overshadowed by antibiotics in Western medicine, has a well-established history in some Eastern European countries and is now being revitalized as a promising strategy against antimicrobial resistance (AMR). This resurgence of phage therapy is driven by the urgent need for innovative countermeasures to AMR, which will cause an estimated 10 million deaths annually by 2050. However, the emergence of phage-resistant variants presents challenges similar to AMR, thus necessitating a deeper understanding of phage resistance mechanisms and control strategies. The highest priority must be to prevent the emergence of phage resistance. Although phage cocktails targeting multiple receptors have demonstrated a certain level of phage resistance suppression, they cannot completely suppress resistance in clinical settings. This highlights the need for strategies beyond simple resistance suppression. Notably, recent studies examining fitness trade-offs associated with phage resistance have opened new avenues in phage therapy that offer the potential of restoring antibiotic susceptibility and attenuating pathogen virulence despite phage resistance. Thus, controlling phage resistance may rely on both its suppression and strategic redirection. This review summarizes key concepts in the control of phage resistance and explores evolutionary engineering as a means of optimizing phage therapy, with a particular focus on *Pseudomonas* infections. Harnessing evolutionary dynamics by intentionally breaking the spontaneous evolutionary trajectories of target bacterial pathogens could potentially reshape bacterial adaptation by acquisition of phage resistance, unlocking potential in the application of phage therapy.

## 1. Bacteriophages, Phage Therapy, and Phage-Resistant Variants

### 1.1. Bacteriophages

Bacteriophages (phages) are prokaryotic viruses that specifically infect and lyse bacteria. Phages are the most abundant biological entities on Earth, with an estimated total exceeding 10^31^ particles [1]. Ubiquitously distributed in a variety of environments, phages play a significant role in shaping bacterial community dynamics [2,3]. The basic structure of phages consists of a capsid that encloses genetic material and an associated tail. The tail is particularly crucial for host recognition, as it binds specifically to bacterial surface receptors and facilitates genome injection [4]. Phage genomes exhibit considerable diversity, comprising double-stranded DNA (dsDNA), single-stranded DNA, and RNA. dsDNA-tailed phages have traditionally been classified into three major families based on tail morphology: Myoviridae (contractile tail), Siphoviridae (long, non-contractile tail), and Podoviridae (short tail). Notably, the majority of phages used for therapeutic applications belong to these morphotypes [5]. However, in 2022, the International Committee on Taxonomy of Viruses abolished the morphological classification scheme for phages and adopted a new system based on genomic information [6].

### 1.2. Phage Therapy

Phages were first discovered by Félix d’Hérelle and Frederick Twort in the early 20th century and initially studied as potential antibacterial agents [7,8]. However, their application declined in Western countries following the discovery of penicillin and subsequent widespread adoption of antibiotics. Nevertheless, interest in phage therapy has recently resurged due to the increasing prevalence of bacteria exhibiting antimicrobial resistance (AMR), which will cause an estimated 10 million deaths annually by 2050 [5,9,10]. Additionally, phage therapy offers a targeted treatment approach that selectively eliminates pathogenic bacteria while minimizing the dysbiosis associated with conventional antibiotics because the host range of phages is specific and limited to a single bacterial genus or species [11,12].

The life cycle of phages exhibits two primary stages: the lytic cycle, in which the host cell is lysed upon phage replication, and the lysogenic cycle, in which the genome of temperate phages is integrated into the host genome to establish a symbiotic relationship. Importantly, integration of the genome of temperate phages can alter bacterial gene expression and facilitate the horizontal transfer of virulence or resistance genes, rendering these phages generally unsuitable for therapeutic applications in their native (wild-type) form. By contrast, strictly lytic (virulent) phages, which efficiently lyse bacteria without integration of the genome, are therefore considered the primary candidates for therapeutic applications [5,13]. However, recent advances in synthetic biology have enabled the engineering of temperate phages to eliminate their lysogenic potential, thereby converting them into synthetic virulent phages suitable for therapeutic applications [14].

### 1.3. Phage-Resistant Variants

A major obstacle to the successful application of phage therapy is the emergence of phage-resistant variants [15,16,17]. These resistant phenotypes commonly arise during phage treatment through alterations, deletions, or modifications of bacterial surface structures—such as outer membrane proteins (omps), lipopolysaccharides (LPS), pili, flagella, and transporters—that serve as phage receptors [18,19,20,21,22], thereby preventing successful phage adsorption. In addition to receptor-based resistance, bacterial sensitivity to phage infection is also shaped by intracellular defense mechanisms. These include the degradation of invading phage genomes via CRISPR-Cas and restriction–modification systems [23], as well as interference with the phage life cycle through the modulation of host cellular processes [24,25]. Collectively, these anti-phage defense systems play a critical role in determining the baseline susceptibility of bacteria to phage infection. Therefore, to effectively manage the emergence of resistance during phage therapy, it is essential to deepen our understanding of the molecular mechanisms underlying resistance, particularly those involving receptor modifications.

In this review, resistance specifically refers to the acquisition of traits that enable a pathogenic bacterium to withstand therapeutic concentrations of a phage (or antibiotic), as distinct from intrinsic insusceptibility.

## 2. *Pseudomonas* Infections Associated with AMR

### 2.1. Pseudomonas aeruginosa

*Pseudomonas aeruginosa* is a rod-shaped, Gram-negative, γ-proteobacterium of the family *Pseudomonadaceae* [26]. The bacterium is a facultative aerobe capable of both aerobic and anaerobic respiration and renowned for exceptional metabolic versatility, evidenced by its ability to utilize a wide range of organic compounds. Due to its adaptability, *P. aeruginosa* can persist in diverse environments, including soil, water, vegetation, and human-associated niches such as the skin and mucosal surfaces [27,28]. *P. aeruginosa* is frequently isolated from hospital reservoirs such as sinks, humidifiers, and respiratory equipment, contributing to its role as a major cause of nosocomial infections. Clinically, *P. aeruginosa* is a prototypical opportunistic pathogen and leading cause of both acute and chronic infections in individuals with underlying conditions such as cystic fibrosis, burn injuries, cancer, chronic obstructive pulmonary disease, and ventilator-associated infections [28,29].

The virulence of *P. aeruginosa* is mediated by a complex array of factors that facilitate tissue invasion, immune evasion, and biofilm formation, which often results in persistent infections with high morbidity and mortality rates, reaching 40% in some populations [26,28]. The high level of intrinsic resistance to many classes of antibiotics and the bacterium’s extraordinary capacity to acquire new resistance mechanisms represent critical concerns in the management of *P. aeruginosa* infections [30]. As such, *P. aeruginosa* is one of the most challenging pathogens affecting clinical practice, making it a priority target for alternative therapeutic strategies, including phage therapy [5,13,31].

### 2.2. AMR in Pseudomonas Infections

The high level of intrinsic insusceptibility of *P. aeruginosa* limits the efficacy of multiple antibiotics, including carbapenems and third-generation cephalosporins. This insusceptibility is primarily attributed to factors such as a low-permeability outer membrane, constitutively expressed efflux pumps, and antibiotic-modifying enzymes [29,30]. Additionally, acquired resistance can arise through chromosomal mutations, which can lead to the overexpression of these intrinsic efflux pumps, or the acquisition of mobile genetic elements, such as plasmids and integrons [29,32]. Acquired resistance produces a variety of phenotypes, such as multidrug-resistant (MDR), extensively drug-resistant (XDR), or pan-drug-resistant (PDR) [29,33]. Given its advanced resistance mechanisms, the World Health Organization classifies *P. aeruginosa* as a high-priority organism on the “priority pathogens list”. Furthermore, *P. aeruginosa* is included in the “ESKAPE pathogens” group along with *Enterococcus faecium*, *Staphylococcus aureus*, *Klebsiella pneumoniae*, *Acinetobacter baumannii*, and *Enterobacter* spp., pathogens considered high-priority targets in the development of new antibiotics [34].

The global prevalence of antimicrobial-resistant *P. aeruginosa* strains continues to increase. According to a 2021 European Centre for Disease Prevention and Control report, 18.7% of *P. aeruginosa* isolates examined were carbapenem-resistant, and 13.4% of isolates were resistant to three or more classes of antibiotics [35]. Moreover, reports of carbapenem-resistant *P. aeruginosa* in the veterinary and agricultural sectors are increasing [36], indicating that the incidence of AMR is also increasing in fields other than human medicine.

The complexity of treating bacterial infections is shaped by multiple clinical and pharmacological factors, not solely by the pathogen’s AMR profile. Patient-specific characteristics, such as allergies or comorbidities, can constrain prescription decisions. Likewise, the ability of an antimicrobial to diffuse to the site of infection at an effective concentration is a critical determinant of therapeutic success. Indeed, many infections are considered “difficult-to-treat” even when caused by strains fully susceptible to tested antibiotics. It is within this multifaceted clinical landscape that the emergence of difficult-to-treat resistant (DTR) *P. aeruginosa* strains in recent years has added another significant layer of complexity to treatment strategies. These strains are resistant to nearly all commonly used antibiotics, including carbapenems, cephalosporins, and quinolones, although they remain susceptible to colistin and novel beta-lactam–beta-lactamase inhibitor combinations such as ceftolozane-tazobactam and ceftazidime-avibactam [37,38]. The Infectious Diseases Society of America introduced the concept of DTR *P. aeruginosa* to highlight strains that present with a complex resistance profile with limited treatment options, distinguishing these strains from MDR and XDR strains. DTR *P. aeruginosa* is primarily isolated in hospital settings, particularly in intensive care units, where excessive antibiotic use and prolonged hospital stays contribute to the selection of DTR strains. The spread of DTR *P. aeruginosa* strains emphasizes the critical need for tailored treatment approaches and stringent antimicrobial stewardship practices to prevent further dissemination and mitigate the impact of resistant organisms on patient outcomes [39].

## 3. Prevention of Phage Resistance Using Phage Cocktails

In the context of the global spread of antimicrobial-resistant *P. aeruginosa*, increasing international interest has focused on the clinical application of phages [5,13,29]. However, pathogenic bacteria can readily acquire phage resistance, notably through receptor alterations [15,17]. Indeed, in vitro experiments examining co-culture of various phages and target *P. aeruginosa* strains demonstrated that bacterial regrowth can occur within a few hours after phage inoculation [40,41], suggesting that phage resistance emerges rapidly, potentially through genomic mutations or, in certain bacterial species, via epigenetic mechanisms in receptor-encoding genes [20,22,42,43]. The phenomenon of phage resistance bears striking resemblance to the historical emergence of antibiotic-resistant bacteria, indicating that development of proactive strategies aimed at anticipating and mitigating phage resistance will be essential for sustainable phage therapy. In the absence of such strategies, phage therapy may follow the same trajectory as antibiotic therapy; that is, initial effectiveness followed by loss of efficacy due to the emergence of resistant bacteria. The control of phage resistance thus represents one of the most critical obstacles to the widespread adoption of phage therapy.

Among possible strategies to overcome phage resistance, the most fundamental and essential involves the early prevention or suppression of resistance emergence. A commonly adopted approach to prevent or suppress phage resistance involves the use of phage cocktails—combinations of multiple phages that target distinct bacterial surface receptors—to reduce the likelihood of resistance development [5,13,15]. This strategy is also applicable to *P. aeruginosa*, and a prevailing principle for effective cocktail design is that each component phage should exploit a different bacterial receptor. However, this principle warrants careful consideration as the definition of a “different” receptor is complicated. For instance, even within a single receptor class like LPS, individual phages may recognize distinct moieties, such as the O-antigen versus the core oligosaccharides, suggesting resistance caused by O-antigen truncation to one may not confer resistance to the other. Furthermore, resistance often arises not from the complete deletion of a receptor but from other mechanisms, such as modified receptor structures or reduced expression levels. These alterations might prevent recognition by one phage while still permitting binding by another that targets the same protein class. Consequently, the acquisition of resistance to a single phage does not necessarily lead to cross-resistance against other phages targeting the same nominal receptor. Indeed, some studies suggest that simultaneous predation by multiple phages can lead to the selection of mutants that circumvent this design logic [44]. Nevertheless, these complexities do not invalidate the cocktail strategy. Instead, they underscore the need for a more sophisticated understanding of phage-host interactions to guide cocktail formulation. This deeper insight could enable the design of cocktails that more potently suppress resistance or even “steer” bacterial evolution toward a clinically manageable state, highlighting a rich area for future investigation. Accordingly, identifying the receptor genes utilized by *P. aeruginosa* phages serves as the rational starting point for such design. Previous studies have shown that phages targeting *P. aeruginosa* exploit multiple receptors, including cell surface LPS—particularly the O-antigen and core oligosaccharides [20,45,46], outer membrane transporters [47], and type IV pili [48,49]. These findings strongly suggest that combining phages that target different classes of receptors, such as LPS and outer membrane proteins, could substantially suppress the emergence of phage resistance in *P. aeruginosa*. Empirical evidence suggests this hypothesis applies to other Gram-negative bacteria as well. For example, the emergence of phage resistance was significantly suppressed in vitro using a combination of phages that target flagella and LPS [50] in *Cronobacter* and phages targeting the O-antigen, BtuB, and OmpC in *Salmonella* [51]. Notably, whereas in vitro co-culture with individual phages (ΦCR8 and ΦS13) allowed the emergence of resistant *Cronobacter* mutants within 5 h, their combination as a cocktail delayed bacterial regrowth for over 11 h [50]. Moreover, in silico modeling studies have demonstrated that cocktails composed of *Pseudomonas*-targeting phages that recognize distinct receptors reduce the probability of simultaneous acquisition of resistance to each phage [52]. A comprehensive study further revealed that resistance of *P. aeruginosa* to 27 different phages exhibited a modular network pattern [53]. In this network structure, phages within the same module tended to exhibit similar resistance profiles, whereas cross-resistance was less likely between modules. These modules corresponded to genetic mutations in distinct receptor-associated genes, such as those related to LPS or type IV pili, indicating that the evolution of phage resistance is strongly associated with phage receptor specificity. Paradoxically, this insight hints at a strategic advantage for phage therapy: phage combinations that target distinct modules (i.e., different classes of receptors) would likely be the most effective for minimizing the risk of resistance development. In support of this hypothesis, recent studies have suggested that combinations of genetically diverse *P. aeruginosa*-targeting phages demonstrate enhanced cocktail effects [54], thus further supporting the receptor-based cocktail design principle. Nevertheless, a recent study using *Pseudomonas alcaligenes*, a close relative of *P. aeruginosa*, reported that sequential administration of individual phages was more effective at preventing the emergence of resistance than application of a multi-phage cocktail [55]. This finding suggests that the optimal dosing protocol—simultaneous versus sequential administration—remains an open question that warrants further investigation and standardization. There is clearly room for refinement in phage therapy strategies aimed at suppressing the emergence of resistant bacteria.

## 4. Phage Resistance in Clinical *Pseudomonas* Infections

The administration of phage cocktails as a strategy to mitigate the challenge of phage resistance has been extensively explored (Table 1). Indeed, five cases of *P. aeruginosa* infection treated at the University of California San Diego between 2018 and 2020 involved the use of phage cocktails for diverse conditions, including post-transplant pneumonia, cystic fibrosis, and ventricular assist device (VAD) infections (Table 1, Cases 4–6) [56,57]. While these therapies, predominantly administered with antibiotics, led to successful outcomes like bacteremia resolution, recurrence was observed in VAD-related infections, potentially due to poor phage penetration into biofilms. The efficacy of combined phage and antibiotic therapy was also demonstrated in other cases of *P. aeruginosa* infection, including those involving ventilator-associated pneumonia (VAP), prosthetic knee infection and Kartagener syndrome (Table 1, Cases 3, 9, 14, and 25) [58,59,60,61]. In addition, case reports of phage cocktail therapy for *P. aeruginosa* infections continue to accumulate. A notable example is a large Belgian-led cohort study, where *P. aeruginosa* was the most frequently treated pathogen (49 of 100 cases), utilizing various phage combinations or predefined cocktails (Table 1, Cases 15–23) [62,63,64,65,66]. Numerous other reports highlight successful outcomes across different clinical contexts. These include the local administration of a single phage with ceftazidime for a chronic infection (Table 1, Case 1) [67], and the use of cocktails to treat a refractory infection with a bronchopleural fistula (Table 1, Case 10) [68], MDR *P. aeruginosa* infection (Table 1, Case 11) [69], and a pediatric case (Table 1, Case 2) [70]. Further successes have been documented in treating a recurrent left ventricular assist device (LVAD)-related infection, where adjunctive surgery was also performed, and a postoperative infection following a Bentall procedure (Table 1, Cases 13 and 26) [71,72]. Conversely, cases in which phage therapy has exhibited suboptimal or limited efficacy have also been reported. In one case of a vascular graft infection, bacteremia recurred despite combined therapy; isolates from the recurrence showed both phage resistance and enhanced biofilm formation (Table 1, Case 12) [73]. Similarly, the emergence of phage-resistant variants was implicated in the treatment failure of a chronic LVAD infection (Table 1, Case 24) [57,74]. Analysis of cases from the Belgian cohort revealed that single nucleotide polymorphisms or deletions in phage receptor genes were the presumed cause of resistance, preventing pathogen eradication (Table 1, Cases 15–17) [62]. Interestingly, however, two of these patients showed clinical improvement despite the failure to eradicate the pathogen, suggesting benefits beyond bacterial clearance (Table 1, Cases 16 and 17).

Common factors that contribute to treatment failure in challenging cases such as those described above include suboptimal delivery of phages to the infection site, rapid emergence of phage resistance, and associated bacterial phenotypic shifts, particularly with regard to increased ability to form biofilms. Notably, the acquisition of phage resistance appears to critically affect the chances of therapeutic success in challenging cases. Furthermore, the diminishment of therapeutic efficacy due to the emergence of phage resistance, even with the use of phage cocktails, is not confined to *P. aeruginosa* but also observed in phage therapy for the treatment of other bacterial pathogens [16,75,76,77]. The potential emergence of phage resistance thus underscores the necessity of developing strategies that not only fundamentally curtail the development of phage resistance but also effectively manage phage-resistant bacterial populations. Moreover, the PhagoBurn clinical trial serves as a critical reminder that beyond these biological and clinical hurdles, ensuring the therapeutic efficacy of phage cocktails also depends heavily on pharmaceutical factors such as their optimal design and long-term stability [78].

## 5. Fitness Trade-Offs with Phage Resistance

The Russian-American geneticist Theodosius Dobzhansky (1900–1975) famously stated, “Nothing in biology makes sense except in the light of evolution”. This evolutionary perspective is profoundly relevant to infection control strategies involving phage therapy. Specifically, this perspective underscores the principle that any approach to managing infections, particularly phage therapy, that disregards evolutionary dynamics is unlikely to achieve sustained and effective therapeutic outcomes. The “fitness” of bacteria (i.e., their capacity to adapt to a given environment) is continually enhanced through the accumulation of diverse phenotypic variations and adaptations essential for survival. The interactions between phages and bacteria represent an integral component of this adaptive process, in which both entities are locked in a perpetual “evolutionary arms race” [79,80,81]. The acquisition of specific traits frequently incurs biological costs that in turn lead to “evolutionary constraints” or “fitness costs”, with the enhancement of one function potentially coming at the expense of another. Such trade-offs are a universally observed phenomenon in biological evolution [82,83]. Notably, recent research revealed that so-called “fitness trade-offs” associated with the acquisition of phage resistance—the ability to evade phage infection—can paradoxically lead to an attenuation of bacterial virulence or reductions in antibiotic resistance levels in antimicrobial resistant bacteria [15,17,83,84]. These fitness trade-offs represent new avenues for phage therapy. Consequently, phage therapy should not be conceptualized solely as a “bactericidal tool” for eliminating pathogenic bacteria but also as a potent “selective pressure” capable of affecting the trajectory of bacterial evolution. Therapeutic strategies employing phages must therefore consider not only the direct lytic activity of the therapy but also the broader evolutionary implications, including potential decreases in pathogenicity and antibiotic resistance stemming from the development of phage resistance. In essence, the infection site serves as a dynamic stage upon which phage–pathogen interactions unfold on an evolutionary timescale. A holistic viewpoint that encompasses this complex interplay is paramount to devising and implementing sustainable infection control strategies.

### 5.1. Fitness Trade-Offs Between Phage Resistance and Bacterial Virulence: Attenuating Virulence

**Figure 1:**
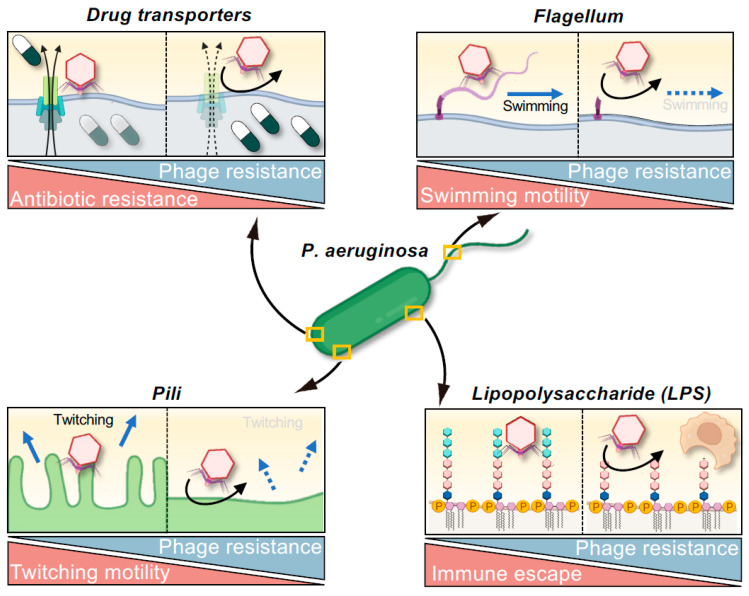
Evolutionary trade-offs associated with phage resistance in *Pseudomonas aeruginosa*. The acquisition of phage resistance can compromise bacterial traits such as antibiotic resistance, motility, and immune evasion. Resistance mechanisms that affect pili or flagella often impair bacterial twitching or swimming motility. Modifications to outer membrane structures, including LPS and drug transporters, can reduce the capacity for immune evasion or increase antibiotic susceptibility. These trade-offs offer therapeutic opportunities by attenuating virulence and reversing AMR, even when phage-resistant variants emerge.

The pathogenicity of *P. aeruginosa* is determined by an intricate pathway involving adhesion to host cells, tissue invasion, and immune evasion. The virulence of *P. aeruginosa* is often mediated by bacterial transmembrane proteins, cellular appendages such as flagella and pili, and polysaccharides such as LPS [29]. Increasing evidence suggests that these virulence factors serve as receptors for phage infection [20,45,46,49]. Consequently, the development of resistance to these phages can lead to an “evolutionary trade-off” characterized by attenuated virulence, and numerous instances of this phenomenon have been reported (Figure 1 and Table 2).

Type IV pili are key virulence factors of *P. aeruginosa*, and they have been implicated as mediating twitching motility and adhesion to host cells. Resistance to type IV pili-targeting phages can result from mutations in genes such as *pilB* or *pilT*, which leads to diminished twitching motility [49,85,86,87,88,89]. Similarly, strains in which flagella-encoding genes such as *flgC* or *motABCD* have been knocked out exhibit a loss of swimming motility [90,91]; this change can impact interactions with host immunity, such as the evasion of released neutrophil extracellular traps [90]. Furthermore, biofilm formation is crucial for the establishment of chronic infections and protection against environmental stress, and the acquisition of resistance to phages that target pili, flagella, or LPS is consistently associated with a diminished capacity for biofilm formation [46,85,89,91,92]. This diminished capacity to form biofilm is often attributed to the loss of motility or changes in surface polysaccharide structures necessary for biofilm formation. Moreover, *P. aeruginosa* strains that have developed resistance to LPS-targeting phages through mutations in the *waaL* gene (which encodes O-antigen ligase) are more susceptible to phagocytosis by bone marrow-derived dendritic cells [91]. This exemplifies a concept known as “immunophage synergy”. Indeed, Roach et al. demonstrated that although treatment with phages alone is sometimes ineffective, successful infection control can be achieved through synergism with host immunity, particularly with regard to neutrophil activation [93], which has clinically significant implications.

These observations reveal a strategic advantage for phage therapy: when phages target virulence structures, the evolution of phage resistance can inherently lead to attenuated pathogenicity. From a clinical standpoint, the prospect of resistant bacterial strains exhibiting debilitated infectivity offers a promising paradigm for future therapeutic design, often referred to as an “evolutionary attenuation strategy” or leveraging “evolutionary trade-offs” in phage therapy. This principle may be reflected in Cases 16 and 17 summarized in Table 1, in which clinical improvement was noted despite the failure to eradicate the target *P. aeruginosa* strain and the suspected emergence of phage-resistant variants [62], suggesting that the resistant bacteria may have become less virulent.

### 5.2. Fitness Trade-Offs Between Phage Resistance and Antibiotic Sensitivity: Reversing AMR

Drug efflux pumps are prominent components of resistance mechanisms in MDR bacteria that reduce the antibiotic susceptibility of a bacterium by actively exporting antimicrobial agents from the cell [29]. In *P. aeruginosa*, the MexAB-OprM and MexXY-OprM efflux systems are particularly significant in this regard, contributing to resistance against a broad spectrum of antibiotics [94,95]. Intriguingly, these efflux pumps can also serve as receptors for certain phages. Consequently, when phages target these structures, bacteria may develop phage resistance through mutations in the genes encoding the pump components or downregulation of their expression. This can result in impaired drug efflux and restoration of antibiotic susceptibility, illustrating compensatory changes associated with adaptive evolution (Figure 1 and Table 2). A prime example of this phenomenon is phage OMKO1, which infects *P. aeruginosa* via OprM, an outer membrane protein common to both the MexAB-OprM and MexXY-OprM efflux systems [47]. Strains of *P. aeruginosa* resistant to ΦOMKO1 reportedly exhibit up to 50-fold increased susceptibility to various antibiotics, including ciprofloxacin, tetracycline, ceftazidime, and erythromycin. Such an increase in antibiotic susceptibility has been corroborated using in vitro studies and *Galleria mellonella* infection models [47,96]. Clinically, the case of a patient with a vascular graft infection treated with a combination of phage OMKO1 and ceftazidime (Table 1, Case 1) suggested that the therapeutic efficacy of ceftazidime was in part attributable to antibiotic re-sensitization due to phage resistance, in addition to direct phage-mediated bacterial lysis [67].

A recently identified phage, PIAS, was found to target the *P. aeruginosa* transporter protein MexY, which is a core component of the MexXY-OprM drug efflux system implicated in the efflux of aminoglycosides and fluoroquinolones [97]. Resistance to ΦPIAS often involves mutations or deletions in the *mexY* gene, leading to marked bactericidal effects when phage therapy is combined with administration of antibiotics such as fosfomycin, gentamicin, tetracycline, and ceftazidime. Sensitization of *P. aeruginosa* to antibiotics can also occur even when phages do not directly target efflux pumps, as large-scale deletions of genomic regions adjacent to efflux pump genes can indirectly compromise efflux system functionality [98]. In this case, resistance of *P. aeruginosa* isolate Pa12 to phage ΦS12-3 was found to be associated with a large chromosomal deletion encompassing the *galU* gene (designated BigD: bacteriophage-induced *galU* deficiency), which resulted in concomitant loss of the MexXY efflux pump and consequently increased susceptibility to fluoroquinolones such as levofloxacin, enrofloxacin, and orbifloxacin. Thus, therapies that promote phage-induced deletions in chromosome could potentially restore the clinical utility of previously ineffective antibiotics [98,99,100,101].

Paradoxically, evolution guided by phage infection can confer the therapeutic benefit of antibiotic re-sensitization. Increased attention is focusing on this “evolutionarily steered therapeutic strategy” as an innovative approach to combat MDR bacteria. Additionally, if mutations exist within the quinolone resistance-determining region (QRDR), the resulting loss of efflux pump function and subsequent intracellular drug accumulation may not restore quinolone susceptibility [99]. Nevertheless, recent case reports have documented instances in which phage resistance has led to the reversion of QRDR mutations to susceptible genotypes (Table 1, Case 17) [62], thereby suggesting avenues for further exploration. Consequently, continued characterization of phages and the development of evolutionarily informed therapeutic strategies are highly anticipated. In addition, a series of personalized inhaled phage therapies strongly supports phage-driven trade-offs in clinical cases, which might affect clinical endpoints [102].

**Table 2:** Expected trade-offs associated with phage resistance in *Pseudomonas aeruginosa*: virulence attenuation and antibiotic resensitization.

Expected Trade-Offs in Phage Resistant *P. aeruginosa*		Reported or Potential Phages	Associated Genes	Remarks	References
Attenuating virulence	Twitching motility	Pili-targeting *Pseudomonas* phages	*pilA*, *pilT*, *pilB*, *pilZ*, *pilO*, *pilN*, *pilY1*, *pilX*, *pilM*, *pilR*	Motility reduction accompanying phage resistance.	[49,85,86,87,88,89]
	Swimming motility	Flagella-recognizing *Pseudomonas* phages	*flgC*, *motABCD*	Reduced motility, potentially aiding evasion of neutrophil NETs.	[90,91]
	Biofilm formation	LPS-recognizing *Pseudomonas* phages	*wzy*	Phage resistance linked to decreased biofilm formation.	[46]
		Flagella-recognizing *Pseudomonas* phages	*motABCD*	Flagellar inactivation leads to reduced biofilm formation.	[91]
		Pili-recognizing *Pseudomonas* phages	*pilT*, *pilB*, *pilO*, *pilN*, *pilY1*, *pilX*, *pilM*, *pilR*	Reduced biofilm production accompanying phage resistance.	[85,89,92]
	Phagocytosis	LPS-recognizing *Pseudomonas* phages	*waaL*	Truncated LPS enhances phagocytosis by mouse BMDCs.	[91]
Reversing AMR	Ciprofloxacin sensitivity	OprM-recognizing *Pseudomonas* phages	*oprM*	Resistance to ΦOMKO1 leads to efflux pump loss and antibiotic sensitization.	[47]
	Tetracycline sensitivity				
	Ceftazidime sensitivity				
	Erythromycin sensitivity				
	Levofloxacin sensitivity	LPS-recognizing *Pseudomonas* phages	Bacteriophage-induced *galU* deficiency (BigD) regions, including *mexX* and *mexY.*	Phage resistance via chromosomal deletion enhances susceptibility to quinolones and other antibiotics but may concurrently promote biofilm formation.	[85,98,99,100]
	Orbifloxacin sensitivity				
	Enrofloxacin sensitivity				
	Colistin sensitivity				[101]
	Tetracycline sensitivity	MexY-recognizing *Pseudomonas* phages	*mexY*	*mexY* mutations or Brmts induced by MexY-targeting phages confer antibiotic susceptibility.	[97]
	Fosfomycin sensitivity				
	Ceftazidime sensitivity				
	Gentamicin sensitivity				
	Quinolone sensitivity	Unknown (not identified in detail)	Quinolone resistance determining region (QRDR), H87D conversion.	Phage-resistant *P. aeruginosa* clinical isolates exhibit QRDR mutation conversion after phage therapy.	[62]

### 5.3. Concerns Regarding Negative Trade-Ups and Unintended Consequences

While a fitness trade-off strategy can be helpful, it also carries potential risks. Previous studies have suggested that if we only look at the beneficial parts of evolution, we might miss possible weaknesses. For instance, large chromosomal deletions caused by phage infection can lead to undesirable phenotypic changes, such as enhanced biofilm formation in *P. aeruginosa* [85]. This emphasizes the critical need for attention, as some phage resistance may drive a negative “trade-up” that increases bacterial resistance to one or more antibiotics, instead of the desired trade-off [17,98]. A notable example involves the coliphages T6 and U115, which target the Tsx porin. This porin is also the entry point for the antibiotic albicidin. Consequently, bacteria that evolved resistance to these phages by mutating *tsx* also exhibited cross-resistance to albicidin, which is a clearly unfavorable “trade-up” in clinical settings [103]. It has also been reported that, while a majority of mutants resistant to the TolC-targeting coliphage U136B became more susceptible to antibiotics, as TolC functions as a drug efflux transporter, a few variants were observed to become more drug-resistant, likely due to synergistic pleiotropy [104]. These highlight that accurately predicting phage-driven evolutionary trajectories remains a significant challenge. Therefore, to refine our predictions for phage therapies and to minimize unintended effects, a deeper understanding of these intricate interactions is essential.

## 6. Core Molecular Machinery Involved in the Acquisition of Bacterial Phage Resistance

The concept of spontaneous symmetry breaking, originally proposed in the context of theoretical physics, refers to the spontaneous transition of a system that is symmetric in its overall structure to an asymmetric state without any explicit external bias [105,106]. A commonly used analogy involves a round table with four glasses placed equidistantly. Although each glass is equally accessible, once the first individual reaches for the glass on their left, other individuals are likely to follow suit, resulting in a collective preference for one side. Despite the absence of any predetermined bias, symmetry is thus “spontaneously” broken. This concept offers a compelling analogy for understanding the evolutionary responses of pathogenic bacteria to phage predation.

In principle, a wide variety of genetic routes could enable bacteria to acquire phage resistance—including changes in the structure of LPS or membrane transporters, or dysregulation of transcriptional regulators [18,42,47]. Even within a single resistance pathway, such as LPS modification, multiple mutations—differing in the genes involved or specific nucleotide changes—can give rise to functionally similar outcomes [20,22,98]. In practice, however, the emergence of a particular mutation often leads to its rapid dominance within the population, resulting in a highly skewed distribution of resistance patterns. This emergent asymmetry—despite a theoretically “symmetric” landscape of mutational possibilities—can be viewed as a form of spontaneous breaking of the symmetry of evolutionary selection. In this sense, the concept of spontaneous evolutionary symmetry breaking in pathogenic bacteria could serve as a framework for understanding how selective pressure channels the adaptive trajectories of bacterial populations. Importantly, such evolutionary shifts do not always result in beneficial outcomes from a clinical standpoint, such as trade-offs that increase antibiotic susceptibility or reduce virulence (Table 2). Indeed, spontaneous evolutionary symmetry breaking can occasionally yield bacterial variants exhibiting enhanced antibiotic resistance and adaptability, thereby complicating infection control efforts [85,98,104]. To address this challenge through strategic design of phages or phage cocktails—or by externally manipulating key stages of the phage infection process—it may be possible to intentionally bias the evolutionary trajectory of a bacterial population towards desired clinical outcomes. This approach represents a form of evolutionary engineering that could potentially reshape bacterial adaptation by acquisition of phage resistance. In the following sections, we illustrate this concept by examining the molecular mechanisms of mutation acquisition and DNA damage repair in relation to the acquisition of phage resistance. Based on these findings, we propose a working model that outlines how the molecular machinery of DNA repair could be deeply involved in the evolutionary trade-offs associated with acquiring phage resistance.

### 6.1. Phage Resistance via the DNA Damage Response and Chromosomal Rearrangement

**Figure 2:**
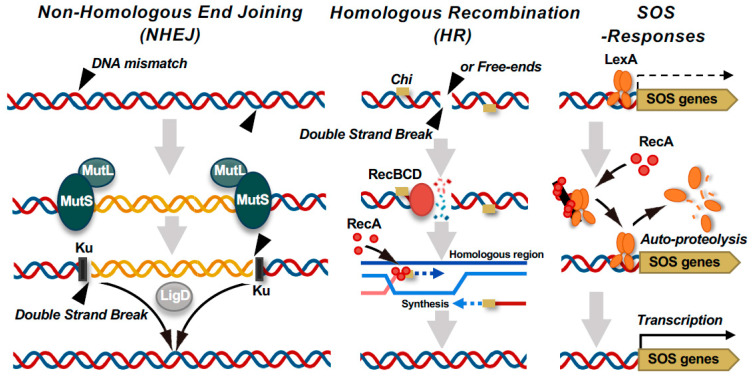
DNA double-strand break (DSB) repair pathways and SOS response activation in bacteria linked to phage resistance. Bacteria utilize multiple pathways to repair DSBs. The non-homologous end joining (NHEJ) pathway involves Ku and LigD, which directly ligate DNA ends, whereas MutS–MutL appears to suppress aberrant recombination. By contrast, homologous recombination (HR), which is initiated by the RecBCD complex, processes DNA ends and facilitates RecA-mediated strand invasion. Notably, RecBCD also functions as a defense factor by recognizing and degrading foreign linear DNA, such as DNA of injected phage genomes. RecA activation leads to LexA autocleavage, triggering the SOS response and inducing genes involved in DNA repair and damage tolerance.

In *P. aeruginosa*, phage resistance can be acquired through large-scale chromosomal deletions [85,97,98,100,101,107,108], mediated by the DNA mismatch repair system, subsequent double-strand breaks (DSBs), and their associated repair mechanisms (Figure 2). Two key mismatch repair components, MutS and MutL, preserve genomic integrity by detecting and correcting misincorporated nucleotide pairs during DNA replication. The recognition of mismatches by MutS and the formation of a MutS-MutL complex initiate localized nucleotide excision, which can go on to form DSBs [109,110,111]. DSBs are repaired through two major pathways: homologous recombination (HR), primarily mediated by RecA and RecBCD, and non-homologous end joining (NHEJ), mediated by Ku and LigD [112,113,114,115]. In the HR pathway, RecBCD unwinds DNA from the DSB ends, and upon recognition of the Chi sequence (5’-GCTGGTGG-3’ in *Escherichia coli*) [116], its mode of activity switches to facilitate RecA loading and homologous strand exchange. This mechanism enables high-fidelity repair and restoration of the original sequence. When HR predominates, the emergence and selection of deletion mutants are relatively suppressed, even under phage pressure. Conversely, the NHEJ pathway directly ligates non-homologous DNA ends through the activity of Ku and LigD, which often results in deletions or insertions. This error-prone repair mechanism frequently leads to the loss of extensive chromosomal regions, including those encoding phage receptors, thereby conferring phage resistance. Under selective pressure from phage infection, mutants harboring such large-scale deletions are likely to be positively selected. Indeed, Shen et al. reported that overexpression of *mutL* in *P. aeruginosa* PAO1 dramatically increases the frequency of mutants with large chromosomal deletions. Similarly, overexpression of *ku* or *ligD* increases the likelihood of such deletions. Moreover, deletion of *recA*—thus disabling the HR pathway—also markedly upregulates the selection of large-scale deletion mutants [107]. Collectively, these findings suggest that the modulation of the dynamics of DNA repair plays a crucial role in shaping bacterial evolutionary responses to phage predation, particularly in promoting resistance via genome restructuring.

### 6.2. RecA-Mediated Mutagenesis: Hypermutable State and the SOS Response

Loss-of-function mutations in MutL or MutS compromise the repair of replication errors, thus driving bacteria into a hypermutable state. In this hypermutable state, point mutation accumulation accelerates markedly, leading to the emergence of diverse resistance mutations [117,118]. Notably, when *mutS* harbors mobile genetic elements, external stressors such as phage infection can trigger transposition events that disrupt *mutS* function, resulting in a rapid and extensive increase in point mutations [119]. This mutation-inducing process is tightly regulated by the bacterial SOS response, which is triggered when RecA binds to damaged DNA, resulting in autocleavage of the LexA repressor and induction of a suite of DNA repair and mutagenic genes (Figure 2) [120,121,122,123]. The induced genes include those encoding error-prone DNA polymerases such as Pol IV (*dinB*) and Pol V (*umuDC*), which introduce mutations [124,125]. Classical studies have elucidated the multifaceted roles of RecA in this context. Ennis et al. demonstrated that RecA tightly regulates the mutation frequency of UV-damaged phage λ [126]. They found that even when LexA was non-functional, no mutagenesis occurred in the absence of activated RecA. Conversely, introduction of a constitutively active RecA mutant sustained mutagenesis even in a LexA-deficient background. These findings indicate that RecA not only initiates the SOS response via LexA cleavage but also directly contributes to mutagenesis as a secondary function. Importantly, RecA exhibits extraordinarily high fidelity in recognizing homologous sequences. In vitro studies have shown that RecA can detect even a single base mismatch during the early stages of HR [127]. Mismatches near the 3’-end significantly suppress recombination, suggesting that RecA prevents erroneous DNA repair by blocking strand exchange when the sequence homology is low. RecA thus functions as a “kinetic proofreader” that actively excludes sequences of imperfect homology. Recent structural studies revealed that RecA assesses sequence homology with remarkable precision, even during the homology search phase, without forcibly unwinding dsDNA [128]. Instead, RecA only minimally disrupts the DNA duplex to exert no internal stress while probing for homology within single-stranded DNA. This mechanism likely plays a key role in maintaining genome stability during recombination and DNA repair processes.

In summary, RecA functions not only as the canonical trigger of the SOS response via LexA cleavage but also plays a central role in mutagenesis and the maintenance of genome stability. By coupling precise homology recognition with mutagenic potential, RecA balances two seemingly contradictory activities—introduction of mutations for adaptation versus ensuring genomic stability for survival—and thus serves as an evolutionary hub that finely tunes the bacterial response to environmental challenges.

### 6.3. The Dual Nature of the RecBCD Complex: DNA Repair and Phage Interference

The Rec system, particularly the RecBCD complex, serves not only as a primary DNA DSB repair pathway but also plays a key role in the phage infection defense mechanism. In *E. coli*, RecBCD exhibits two distinct modes of action: a destructive mode, in which the complex acts as a potent nuclease that degrades free DNA ends into single-stranded fragments, and a recombinogenic mode, in which encountering a chi site triggers the loading of RecA and initiation of HR repair (Figure 2) [129]. The destructive mode provides multifaceted protection against phage invasion by degrading phage genomes, supplying protospacer sequences to CRISPR libraries, and suppressing rolling-circle replication [130,131,132]. The recombinogenic mode, by contrast, is activated upon recognition of the chi sequence, which is overrepresented in the host genome but rare in phage DNA. This difference enables RecBCD to distinguish “self” from “non-self”, thus integrating repair and immune functions in a context-dependent manner. Recent studies have revealed that certain phages encode proteins collectively referred to as “anti-RecBCD” factors, which interfere with the activity of the RecBCD complex to enable phages to evade bacterial defenses [133]. However, the broader implications of such phage countermeasures—particularly their impact on RecBCD-mediated functions such as mutagenesis and chromosomal rearrangement—remain largely unexplored. As discussed in this chapter, DNA repair mechanisms, including HR and NHEJ, underpin the molecular evolution through which bacteria acquire resistance to phage infection. Targeting these pathways to deliberately disrupt bacterial spontaneous evolutionary selection could provide a novel conceptual framework for studying the evolution of pathogenic bacteria towards desired clinical outcomes.

### 6.4. The Evolutionary Arms Race and Cooperation Between E. coli and Escherichia-Targeting Phages

While the main focus of this review is on *P. aeruginosa*, the extensively studied evolutionary arms race between the model organism *E. coli* and its phages offers invaluable insights. This relationship serves as a rich source of examples for finely tuned molecular adaptation strategies and provides a crucial framework for understanding the principles of the DNA repair-based working model discussed earlier. Therefore, the interactions between *E. coli* and its phages offer valuable lessons for advancing evolutionary engineering in synthetic biology and for developing intervention strategies applicable to other pathogens like *P. aeruginosa*. The efficiency of T7 phage DNA replication is enhanced through utilization of *E. coli* thioredoxin as an accessory factor to compensate for the low processivity of the phage’s own DNA polymerase [134]. To outcompete the host cell’s RNA polymerase and ensure successful transcription of phage genes, T7 has evolved proteins such as Gp2 and Gp5.7, which sequentially inhibit the host’s transcriptional machinery [135,136]. Furthermore, coordinated interactions between multifunctional proteins—such as T7 RNA polymerase and lysozyme—allow for temporal and spatial regulation of key steps in the phage life cycle, including transcription, replication, and DNA packaging [137]. These molecular interactions and network-level control strategies, which have been forged through co-evolution, can be viewed as products of evolutionary optimization in nature. Recent technological advances have increased the feasibility of inserting new genes into phage genomes or knocking out existing genes [138,139,140]. From an evolutionary perspective, such manipulations effectively “skip” or “rewind” the natural evolutionary processes of phages. By elucidating and then mimicking, and reconstructing these mechanisms, we can uncover essential design principles that will enable the future development of evolution-informed phage therapies. Ultimately, this approach promises to provide powerful insights into how evolution itself can be harnessed and deliberately manipulated for infection control.

## 7. Concluding Remarks

One of the most critical challenges limiting the use of phage therapy to treat antimicrobial-resistant bacteria is the emergence of phage resistance. Although it is essential to first suppress the development of phage resistance, even when resistance does arise, strategies are available to actively exploit evolutionary trade-offs. Specifically, the fitness costs associated with phage resistance can be harnessed to deliberately direct bacterial evolution toward reduced virulence and restored antibiotic susceptibility. Such evolutionary interventions not only contribute to restoring the efficacy of conventional antibiotics but also enhance the sustainability of phage therapy by exploiting fitness costs, thereby providing a foundation for preserving effective treatment options into the future. It is important to acknowledge, however, that the practical application of this framework will probably face challenges: beneficial evolutionary trade-offs may not be universally achievable for all phage-bacteria pairings, and furthermore, the reliable induction of specific resistance mutations that consistently lead to such advantageous trade-offs may not always be feasible. On the other hand, a greater understanding of the evolutionary mechanisms that direct the interplay between pathogenic bacteria and phages would offer more than just a means of overcoming resistance; such an understanding would also pave the way for the development of innovative approaches that enhance the overall impact of phage-based treatments. Ultimately, intentionally breaking the spontaneous evolutionary trajectories of bacterial pathogens to redirect their adaptation may unlock unforeseen frontiers in the design of phage therapies against AMR.

## Figures and Tables

**Table 1:** Clinical cases of phage therapy for *Pseudomonas aeruginosa* infections: therapeutic protocols and outcomes. NA; not applicable.

Case	Reference	Year Reported	Major Pathogen	Patient	Phages	Administration	Outcome	Result	Details	Remarks
1	[67]	2018	*P. aeruginosa*	76-year-old male, post aortic arch replacement surgery with Dacron graft for an aortic aneurysm.	ΦOMKO1 (targets the antibiotic efflux pump of *P. aeruginosa*).	Single administration of phage ΦOMKO1 solution (10^7^ PFU/mL, 10 mL) injected into the mediastinal fistula communicating with the abscess cavity surrounding the graft, combined with ceftazidime (0.2 g/mL).	Following a single combined dose of phage and ceftazidime, the *P. aeruginosa* infection clinically improved, with no signs of recurrence at discharge.	Partial success	At 4 weeks post-treatment, the patient developed an aortic perforation due to adhesions and heterotopic ossification, unrelated to phage therapy, requiring emergency surgery. Intraoperative cultures were negative for *P. aeruginosa* (only *Candida* detected from superficial wounds). Antibiotic treatment was discontinued postoperatively, and no recurrence of *P. aeruginosa* infection occurred during 18 months of follow-up.	ΦOMKO1 binds to the *P. aeruginosa* antibiotic efflux pump, exploiting an evolutionary trade-off in which phage resistance increases antibiotic susceptibility. No direct adverse effects of phage administration were reported.
2	[70]	2018	*P. aeruginosa*	2-year-old male with DiGeorge syndrome and complex congenital heart disease.	Two-phage cocktail derived from US Navy phages.	Intravenous administration of 3.5 × 10^5^ PFU every 6 h, combined with existing antibiotics (meropenem, tobramycin, polymyxin B).	After two courses of phage therapy (the first interrupted temporarily and then resumed), blood cultures became negative within days on both occasions. Clinical and microbiological improvement from a persistent 4-week bacteremia was observed, though imaging could not confirm effects at infection sites.	Partial success	Patient ultimately died of cardiogenic and septic shock. However, blood culture negativity was achieved, indicating a significant therapeutic response.	Phage was approved as an emergency investigational new drug by the FDA.
3	[58]	2019	*P. aeruginosa*	77-year-old female with ventilator-associated pneumonia (VAP) and empyema after lung resection surgery.	AB-PA01 (4-phage cocktail).	Intravenous (1 mL at ~1 × 10^9^ PFU/mL, twice daily) and nebulized (4 mL at ~1 × 10^9^ PFU/mL, twice daily) for 7 days, adjunctive to antibiotics.	Infection resolved, leading to pathogen eradication.	Success	The therapy was well-tolerated.	No adverse events reported.
4	[56]	2020	*P. aeruginosa*	67-year-old male lung transplant recipient with recurrent MDR *P. aeruginosa* pneumonia.	Episode 1: AB-PA01 (4-phage cocktail).	4 × 10^9^ PFU/mL intravenously every 6 h and inhalation every 12 h.	Improvement observed with combined phage therapy and antibiotics. Subsequently, phage monotherapy was used for suppressive treatment, with no infection recurrence.	Success	Two distinct pneumonia episodes were resolved by combined phage and antibiotic therapy, followed by phage-only suppressive therapy with no relapse during the suppression period.	No phage-related adverse events reported. Time from phage request to administration was 22 days.
					Episode 2: AB-PA01 m1 (5-phage cocktail) plus Navy phage cocktail 1 (3-phage cocktail).	5 × 10^9^ PFU/mL IV every 6 h and inhalation every 12 h, plus 1 × 10^9^ PFU/mL IV every 2 h and inhalation every 4 h for 4 weeks.				
					Suppressive therapy: Navy phage cocktail 2 (2-phage cocktail).	5 × 10^7^ PFU/mL IV every 4 h for 8 weeks.				
5	[56]	2020	*P. aeruginosa*	26-year-old female with cystic fibrosis and chronic respiratory failure complicated by acute exacerbation of MDR *P. aeruginosa* pneumonia, unresponsive to antibiotics.	AB-PA01 (4-phage cocktail).	5 × 10^7^ PFU/mL intravenously every 4 h for 8 weeks.	No improvement with antibiotics; clinical improvement achieved with phage therapy. No cystic fibrosis exacerbation for 3 months afterward.	Success	Underwent lung transplantation 9 months later. Colistin was discontinued during phage therapy, with resolution of acute kidney injury.	No phage-related adverse events reported. Time from phage request to administration was 23 days.
6	[56]	2020	*P. aeruginosa*	60-year-old male with MDR *P. aeruginosa* driveline infection, complicated by sternal osteomyelitis and recurrent bacteremia for approximately 9 months prior to phage therapy.	GD-1 (3-phage cocktail).	Intravenous infusion of 1.9 × 10^7^ PFU/mL every 8 h for 6 weeks.	Developed bacteremia 1 week after starting phage therapy, which resolved after antibiotic modification. Following completion of phage therapy, recurrent driveline drainage occurred.	Failure	Reinfection occurred, requiring surgical intervention.	No phage-related adverse events reported. Time from phage request to administration was 37 days.
7	[56,57]	2020	*P. aeruginosa*	82-year-old male with persistent ventricular assist device (VAD) infection, multiple hospitalizations over more than 2 years prior to phage therapy, complicated by surgical debridement and recurrent bacteremia.	Episode 1: SDSU1 (2-phage cocktail: PAK_P, E217) and SDSU2 (2-phage cocktail: PAK_P1, PAK_P5).	Intravenous (IV) infusion of 2 × 10^5^ PFU/mL every 8 h for 6 weeks, plus a single intraoperative dose. Subsequently, PAK_P1 alone at 7.58 × 10^5^ PFU/mL for 10 days, followed by SDSU2 at 4 × 10^10^ PFU IV every 12 h for 3 weeks, with piperacillin-tazobactam for days 1–3 and then ceftazidime/avibactam from days 4–50.	On day 4 of phage therapy, breakthrough bacteremia and septic shock occurred. Phage therapy was reinitiated 3.5 months later, but during episode 2, recurrent bacteremia developed at week 4 despite ongoing therapy.	Failure	Isolates after therapy showed significantly reduced susceptibility to PAK_P1 and PAK_P5. Phage stability issues were discussed, with a 4-log titer drop for PAK_P1 and 1-log drop for E217 at therapeutic dilution after 69 days.	Time from phage request to administration was 58 days.
					Episode 2: PPM3 (4-phage cocktail).	IV infusion of 1 × 10^9^ PFU/mL every 12 h for 4 weeks, with ceftolozane/ tazobactam and ciprofloxacin.				
8	[56]	2020	*P. aeruginosa*	64-year-old male with recurrent *P. aeruginosa* bacteremia over the previous 1.5 years, history of prolonged antibiotic courses, and breakthrough infections. Possible aortic graft infection.	PPM2 (3-phage cocktail).	Intravenous (IV) infusion: 2.6 × 10^6^ PFU/mL every 12 h for 6 weeks.	During phage therapy combined with ciprofloxacin, blood cultures remained negative.	Success	After completion of therapy, weekly surveillance blood cultures for 12 weeks (4 consecutive negative cultures) showed no recurrence. Previous bacteremia recurred within 7–10 days after antibiotic discontinuation.	No phage-related adverse events reported. Time from phage request to administration was 374 days.
9	[59]	2021	*P. aeruginosa*	88-year-old male with a relapsing prosthetic knee infection.	Cocktail of 3 phages (PP1450, PP1777, PP1792) selected from the Pherecydes Pharma library.	A single local injection of the phage cocktail (30 cc at 1 × 10^9^ PFU/mL) into the joint via arthroscopy, combined with intravenous ceftazidime and followed by long-term suppressive oral ciprofloxacin.	The patient’s condition improved rapidly, with the disappearance of knee pain. The knee’s local status was normal, and walking was painless during the 1-year follow-up.	Success	This was a salvage therapy for a relapsing infection where standard treatments had failed.	The patient died one year later from unrelated causes, with no clinical signs of the joint infection recurring.
10	[68]	2022	*P. aeruginosa*	68-year-old male with empyema and bronchopleural fistula (BPF) following lung resection. Pneumonia also present.	Cocktail (2 phages).	Administered for 24 days in combination with antibiotics.	Pathogen eradication and clinical improvement were observed.	Success	Suggests that combined phage therapy and conventional antibiotic treatment may be effective for MDR infections.	The treatment was well-tolerated.
11	[69]	2022	*P. aeruginosa*	74-year-old male, undergoing melanoma treatment with anti-PD-1 antibody pembrolizumab. Presented with spinal abscess extending from L2–L3 intervertebral disc to L3–L4 vertebral body.	Phage cocktail (3 phages): vB_PaeP_4029, vB_PaeP_4032, vB_PaeP_ 4034.	Local application during two surgical procedures (final phage titer 10^6^ PFU/mL). After the second surgery, intravenous infusion daily over 3 h for 21 days (30 mL, phage titer 10^6^ PFU/mL). Combined with cefiderocol and colistin.	*P. aeruginosa* was still detected after the first phage plus antibiotic treatment and surgery, but the outcome was favorable after the second treatment course.	Success	Despite bacterial persistence and emergence of small colony variants (SCVs), the patient was cured. SCVs remained sensitive to the phage cocktail. After 21 months of follow-up, no implant loosening or clinical signs of infection were observed. Although *P. aeruginosa* infection was cured, the patient later died due to COVID-19 pneumonia.	No adverse events related to phage therapy. Phage screening, manufacturing, and purification required 3 months, which was a limiting factor.
12	[73]	2023	*P. aeruginosa*	Male in his 50s, vascular graft infection caused by MDR *P. aeruginosa.*	Phage cocktail (3 phages): PT07, 14/01, PNM.	Intravenous infusion once daily, 6 h continuous infusion for 3 days, followed by 4 days of outpatient parenteral antimicrobial therapy.	No therapeutic effect; bloodstream infection recurred after treatment.	Failure	Possible emergence of phage-resistant bacteria after relapse.	NA
13	[71]	2023	*P. aeruginosa*	54-year-old male diagnosed with MDR *P. aeruginosa* LVAD driveline infection 46 months after LVAD implantation, presenting with pus discharge from the anastomosis site, inflammation, fever, and elevated inflammatory markers.	Phage cocktail (2 phages): PT07, PNM.	Administration: intravenous administration starting 2 h before surgery and continuing for 8 days postoperatively at 10^7^ PFU/mL, combined with local administration via catheter along the driveline used intraoperatively and postoperatively to irrigate the surgical wound with phage suspension. LVAD driveline repositioning surgery and systemic antibiotics (ceftazidime/avibactam and amikacin) were also used.	No recurrence for 21 months.	Success	Surgical intervention and multidrug phage therapy contributed to success.	Highlights the importance of adjunctive therapies such as surgical intervention in biofilm-associated infections.
14	[60]	2023	*P. aeruginosa*	41-year-old male with Kartagener syndrome complicated by MDR *P. aeruginosa* infection.	Single phage (vFB297).	Nebulized for 5 days combined with intravenous antibiotics.	Overall clinical improvement. Discontinuation of antibiotic therapy and mechanical ventilation.	Success	No emergence of phage-resistant strains; all bacterial isolates recovered after phage therapy remained phage-sensitive.	NA
15	[59]	2024	*P. aeruginosa* *Staphylococcus aureus*	Chronic upper airway infection with *P. aeruginosa* and *S aureus*, chronic sinusitis.	Cocktail of 3 phages (14-1, PNM, ISP [BFC 1]).	Nasal spray for 10 days; no concurrent antibiotic therapy.	Failure to eradicate target bacteria; emergence of phage-resistant variants.	Failure	Detected a missense mutation in *pilC* (p. Ala154Pro) in the phage-resistant variants, involved in type IV pilus biosynthesis.	No evidence of immune-mediated phage neutralization was found.
16	[62]	2024	*P. aeruginosa*	*P. aeruginosa* infection and COVID-19–associated ventilator-associated pneumonia (VAP).	Cocktail of 3 phages (14-1, PNM, PT07).	Nebulized for 1 week combined with intravenous colistin.	Patient’s overall condition improved, but in vitro assays showed no clear interaction between PNM and 14-1 phages and colistin.	Success	Although the target bacteria were not eradicated and phage-resistant variants emerged, therapeutic effect was observed. Detected a missense mutation in *pilR* (p. Thr230Pro) in the phage-resistant variants, involved in type IV pilus biosynthesis.	No immune-mediated phage neutralization was detected at 2 months post-treatment.
17	[62]	2024	*P. aeruginosa*	Acute *P. aeruginosa* infection with COVID-19–associated acute respiratory distress syndrome (ARDS) and sepsis.	Cocktail of 3 phages (14-1, PNM, PT07).	Phage cocktail combined with intravenous meropenem, colistin, and vancomycin.	Overall clinical improvement; mechanical ventilation was no longer required. In vitro synergy was observed between PT07 and colistin as well as meropenem.	Success	Phage-resistant variants emerged, but therapeutic benefit was achieved. *P. aeruginosa* was still detected at low levels in tracheal aspirates after treatment. Mutations detected in phage-resistant isolates included: LPS biosynthesis-related mutations: *wapH* (p. Trp139X), *galU* (p. Gln239X), *wapR* (p. Leu162Pro), *wbpR* (p. Leu60_Leu63del); type IV pilus-related mutation: *fimV* (p. Arg120fsX, N-terminal 165 amino acid deletion); Others: *cupE5* (p. Gly406Ser), *mexB* (p. Arg994Gly), *gyrA* (p. His87Asp).	No immune-mediated phage neutralization detected at 2 months post-treatment.
18	[62,63]	2024	*P. aeruginosa*	Pediatric male with biliary atresia, developed severe sepsis due to XDR *P. aeruginosa* after living donor liver transplantation.	Cocktail of 3 phages (14-1, PNM, ISP (BFC 1)).	Administration of 10^8^ PFU of BFC1. During the deceased donor liver transplantation, 250 mL of BFC1 was used for intraperitoneal lavage. A total of 86 days of intravenous phage therapy was performed, combined with antibiotics.	Within 36 h of starting phage therapy, two consecutive blood cultures turned negative for *P. aeruginosa*. On day 8, the phage dose was doubled, achieving sustained eradication of *P. aeruginosa* from the bloodstream. Two transient episodes of bacterial translocation from liver abscesses to bloodstream occurred but resolved within 24 h without additional treatment, with clinical stabilization of the patient. Bloodstream infection was controlled by combined phage and antibiotic therapy; however, *P. aeruginosa* persisted in liver lesions.	Success	*P. aeruginosa* was still detected in tissue samples from the explanted diseased liver during re-transplantation. More than 2 years after the second liver transplantation, the patient maintained normal liver function and good clinical condition with no further infections; *P. aeruginosa* colonies were completely eradicated. Among seven *P. aeruginosa* isolates recovered during phage therapy, four showed phenotypic resistance to all phages in the BFC1 cocktail. Whole-genome sequencing revealed genetic mutations in the type IV pilus complex in these resistant variants, known as the adsorption site of phage PNM.	NA
19	[62,64]	2024	*P. aeruginosa* *Staphylococcus epidermidis.*	Chronic pelvic osteomyelitis caused by *P. aeruginosa.* and *S. epidermidis.*	Cocktail of 3 phages (14-1, PNM, ISP (BFC 1)).	7-day administration of phage cocktail via catheter, combined with intravenous vancomycin, oral rifampicin, and oral moxifloxacin for 3 months.	No relapse	Success	Following a single course of phage therapy administered alongside antibiotics, no recurrence of infection with the causative strains was observed during follow-up periods ranging from 8 to 16 months.	No induction of anti-phage antibodies was detected.
20	[62,65]	2024	*P. aeruginosa*	61-year-old male with necrotic pressure ulcer infected with *P. aeruginosa* and associated *P. aeruginosa* septicemia.	Cocktail of 3 phages (14-1, PNM, ISP (BFC 1)).	Local and intravenous administration of phage cocktail for 10 days.	Blood cultures became negative shortly after initiation of phage therapy. Clinical improvements included decreased CRP, defervescence, and recovery of renal function. Blood filtration was avoided.	Success	Treatment of *P. aeruginosa* septicemia was successful; however, the patient died 4 months later due to *Klebsiella pneumoniae* septicemia unrelated to the phage targets. The pressure ulcer remained colonized by bacteria.	No adverse events related to phage therapy were observed.
21	[62,66]	2024	*P. aeruginosa* *Acinetobacter baumannii.*	21-year-old male with chronic osteomyelitis of the femur caused by MDR *P. aeruginosa* and MDR *A. baumannii.*	Cocktail of 3 phages (14-1, PNM, ISP (BFC 1)).	Local phage administration combined with linezolid and ceftazidime-avibactam. Antibiotics continued for 5 months after discharge.	Complete cure without systemic symptoms. No relapse during 1-year follow-up period after treatment.	Success	Eradication of bacteria in the proximal femur was achieved, avoiding lower limb amputation. Nine months after treatment, no signs of inflammation were observed in the proximal femur, allowing total hip arthroplasty to be performed.	No adverse events related to phage therapy were observed.
22	[62]	2024	*P. aeruginosa*	Chronic infection with MDR *P. aeruginosa* and anal fistula.	Cocktail of 3 phages (14-1, PNM, PT07).	Local injection into the lesion; no concurrent antibiotic therapy.	Replacement by a different *P. aeruginosa* strain insensitive to 14-1, PNM, and PT07 was observed, but clinical course remained favorable.	Success	Emergence of phage-resistant bacteria suggested by the appearance of other *P. aeruginosa* clones.	No phage-neutralizing antibodies detected at 4 and 7 months post-treatment.
23	[62]	2024	*P. aeruginosa*	Chronic *P. aeruginosa* infection in cystic fibrosis-associated pulmonary disease.	Cocktail of 2 phages (4P, DP1).	Nebulized delivery; combined with antibiotic therapy.	A different *P. aeruginosa* strain insensitive to 4P and DP1 emerged, but the clinical course remained favorable.	Success	Emergence of phage-resistant bacteria suggested by the appearance of alternative *P. aeruginosa* clones. Synergistic effect observed between 4P/DP1 and levofloxacin; no clear interaction with tobramycin.	No neutralizing effect against phages detected.
24	[57,74]	2024	*P. aeruginosa*	10-year-old female with genetic cardiomyopathy; chronic *P. aeruginosa* infection due to intravascular LVAD infection (persisting for 4 months prior to phage therapy).	Single phage (PASA16).	Combined with meropenem; intravenous infusion at 5 × 10^10^ PFU/mL, twice daily for 51 days.	During treatment, the patient experienced febrile spikes and altered consciousness, though blood cultures remained negative. Clinical deterioration occurred on day 33. Phage and meropenem therapy was discontinued on day 51. Three days later, MDR *P. aeruginosa* bacteremia recurred. The patient died.	Failure	The baseline strain was phage-sensitive. Post-treatment isolates (2/2) showed reduced plaque formation, indicating decreased sensitivity. A newly emerged phenotypically distinct strain (SH3) was also detected.	NA
25	[61]	2024	*P. aeruginosa*	52-year-old male with severe burns (81% of total body surface area) who suffered from relapsing VAP, skin graft infection, and bacteremia.	Cocktail of two phages (PP1792 and PP1797).	Each 7-day course involved combined intravenous (daily) and nebulized inhalation. The therapy was combined with last-resort antibiotics and immunostimulation (interferon-gamma).	The patient improved after the first course but relapsed one month later. Following the second course of phage therapy, the patient was successfully extubated, and the final outcome was favorable, leading to discharge from the ICU.	Success	Phage therapy was used as an adjunct treatment for a relapsing infection that was not controlled by last-resort antibiotics.	No adverse events attributable to phage therapy were reported.
26	[72]	2025	*P. aeruginosa*	58-year-old male with *P. aeruginosa* infection following Bentall procedure.	Cocktail of two myoviruses (PP1450 and PP1777).	Intravenous administration of 10^10^ PFU every 12 h for 7 days. Concurrent antibiotics: ceftazidime and ciprofloxacin for 3 months, followed by ciprofloxacin monotherapy for an additional 3 months.	All blood cultures remained negative after phage therapy, and CRP levels remained negative for up to 12 months post-treatment. Infection was successfully controlled.	Success	Phage therapy was initiated due to failure of antibiotic therapy alone and the high risk associated with surgical intervention.	NA
